# Rising Mediterranean Sea Surface Temperatures Amplify Extreme Summer Precipitation in Central Europe

**DOI:** 10.1038/srep32450

**Published:** 2016-08-30

**Authors:** Claudia Volosciuk, Douglas Maraun, Vladimir A. Semenov, Natalia Tilinina, Sergey K. Gulev, Mojib Latif

**Affiliations:** 1 GEOMAR Helmholtz Centre for Ocean Research Kiel, Kiel, Germany; 2 Wegener Center for Climate and Global Change, University of Graz, Austria; 3 A.M. Obukhov Institute of Atmospheric Physics, Russian Academy of Sciences, Moscow, Russia; 4 Institute of Geography, Russian Academy of Sciences, Moscow, Russia; 5 P.P. Shirshov Institute of Oceanology, Russian Academy of Sciences, Moscow, Russia; 6 Lomonosov Moscow State University, Moscow, Russia; 7 Cluster of Excellence “The Future Ocean”, Christian-Albrechts-Universität zu Kiel, Kiel, Germany

## Abstract

The beginning of the 21st century was marked by a number of severe summer floods in Central Europe associated with extreme precipitation (e.g., Elbe 2002, Oder 2010 and Danube 2013). Extratropical storms, known as Vb-cyclones, cause summer extreme precipitation events over Central Europe and can thus lead to such floodings. Vb-cyclones develop over the Mediterranean Sea, which itself strongly warmed during recent decades. Here we investigate the influence of increased Mediterranean Sea surface temperature (SST) on extreme precipitation events in Central Europe. To this end, we carry out atmosphere model simulations forced by average Mediterranean SSTs during 1970–1999 and 2000–2012. Extreme precipitation events occurring on average every 20 summers in the warmer-SST-simulation (2000–2012) amplify along the Vb-cyclone track compared to those in the colder-SST-simulation (1970–1999), on average by 17% in Central Europe. The largest increase is located southeast of maximum precipitation for both simulated heavy events and historical Vb-events. The responsible physical mechanism is increased evaporation from and enhanced atmospheric moisture content over the Mediterranean Sea. The excess in precipitable water is transported from the Mediterranean Sea to Central Europe causing stronger precipitation extremes over that region. Our findings suggest that Mediterranean Sea surface warming amplifies Central European precipitation extremes.

Observational records show no clear trend in the intensity of summertime heavy rainfall in Central Europe^1^,^2^,^3^,^4^. Yet, climate models generally project an increase of Central European summer precipitation extremes, even though mean precipitation is projected to decrease^5^,^6^,^7^,^8^,^9^. These projected thermodynamic changes are a regional expression of global changes in the hydrological cycle that are primarily caused by increasing saturation vapour pressure of the warming atmosphere^10^,^11^.

Flood producing summer precipitation extremes in Central Europe are often associated with Vb-cyclones^12^,^13^,^14^, southerly west-east cyclone tracks and cut-off lows^15^. Vb-cyclones are typically generated over the Gulf of Genoa, travel northeastward around the Alps and can transport large amounts of moisture towards Central Europe. Persistent cut-off lows over the northern central Mediterranean can cause precipitation extremes in Central Europe similar to those produced by Vb-cyclones. Thus, the Mediterranean Sea serves as a major moisture source for Central European heavy-precipitation events and associated flooding^16^,^17^.

The Mediterranean is itself a hotspot of global warming^18^. Its surface has continuously warmed since the mid–1970s, especially during summer (June, July, August; JJA). In fact, the beginning of the 21st century (2000–2012) featured the highest SSTs in the Mediterranean Sea during the instrumental record^19^ (Fig. 1a). Moreover, the increase from the 1970–1999 to the 2000–2012 period in Mediterranean SSTs (annual mean: 0.62 °C, JJA mean: 0.86 °C) is considerably stronger compared to that of the global oceans (annual mean: 0.15 °C, JJA mean: 0.19 °C). These trends are projected (by the coupled model intercomparison project phase 5; CMIP5-ensemble) to continue throughout the 21st century, with summertime SSTs reaching a warming of more than 2 °C by the end of the 21st century (2071–2098) compared to 1980–2005^20^. Higher Mediterranean SSTs will in turn lead to enhanced evaporation and atmospheric moisture transport^21^, with potential impacts on Central European precipitation. For instance, high Mediterranean SSTs contributed to the extreme precipitation that led to the Elbe-flooding in 2002^16^. Yet, the impact of sea surface warming on such heavy precipitation events cannot be studied in detail based on observations alone due to the relatively short period of recent high SSTs and given the rareness of extreme events.

Summer precipitation extremes in Central Europe may be amplified due to changes in convective storm dynamics (induced by, e.g., changes in vertical atmospheric profiles) or changes in cyclone-related precipitation. For instance, there are two major factors that may contribute to the intensification of cyclone-related summer precipitation extremes in Central Europe: changes in cyclone occurrence or pathway (dynamic changes) and changes in the amount of moisture carried by individual cyclones (thermodynamic changes). Yet, confidence in the thermodynamic aspects of climate change is generally higher than in dynamic changes^22^. The Mediterranean storm track in summer has intensified during recent decades^23^. Climate models do not simulate significant trends in the number of summer cyclones in the Mediterranean region however^24^. Considering Vb-cyclones, no trends have been observed to date in their occurrence^25^, and climate model projections even suggest a slight decrease of Vb-cyclone occurrence^14^,^26^. Nevertheless, the amount of precipitation associated with Vb-cyclones is projected to increase^14^,^26^, raising the question of where the additional precipitable water originates.

## Methods

**Figure 1:**
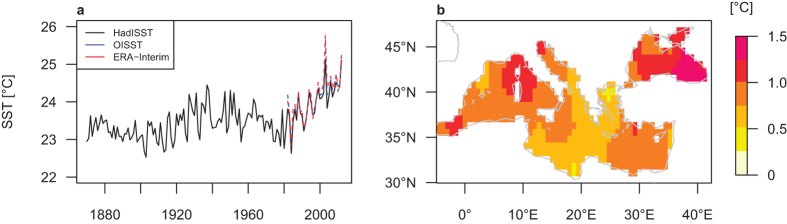
Observed Mediterranean summer SSTs. (**a**) Time series of JJA mean (°C). Area average over 5°W–42°E and 30°N–48°N from HadISST^19^, OISST^41^ and ERA-Interim^34^; (**b**) Significant difference (based on a two-sided independent samples t-test at the 95% significance level) in JJA mean SST patterns (°C) between the periods 2000–2012 (forcing climatology of warm Mediterranean (Med_warm_) experiment) and 1970–1999 (forcing climatology of control experiment) from HadISST^19^. Map created with R version 3.2.3 (www.r-project.org).

In this study, by conducting sensitivity experiments with the atmosphere general circulation model (AGCM) ECHAM5^27^, we aim to disentangle the impacts of Mediterranean Sea warming on Central European precipitation extremes from other effects in the background climate. To this end, we carry out experiments that are identical except for SST and sea ice concentration (SIC). The control experiment is forced globally with monthly climatological fields of 1970–1999 SST and SIC. In the warm Mediterranean experiment (Med_warm_), we employ the warmer 2000–2012 SST climatology only in the Mediterranean and Black Seas (see Fig. 1b for the difference in JJA SST forcing climatology). In the global warm experiment (Glob_warm_), the AGCM is forced globally with monthly climatological fields of 2000–2012 SST and SIC. For each experiment we computed a 40-member ensemble of one year. The model is integrated at a relatively high horizontal resolution for a global atmosphere model of T159 (equivalent to approximately 0.75° × 0.75° lat/lon) and with 31 vertical levels. Forcing conditions for all three experiments were taken from the Hadley Centre Sea Ice and Sea Surface Temperature dataset (HadISST)^19^. Radiative and greenhouse gas forcings are fixed to present-day levels.

We analyse summer precipitation extremes and the associated atmospheric mechanisms in the three model experiments. We define heavy-precipitation events as days where daily precipitation exceeds the 95th percentile of all summer (JJA) days (wet and dry). To have the same sample size for all simulations we individually choose the 95th percentile of all summer days as threshold in each experiment, i.e., in all experiments the 5% of all summer days with the heaviest precipitation are analysed. Differences between the 40-year-long experiments are compared for (i) climatological seasonal summer means over all summer days (wet and dry), and (ii) mean heavy-precipitation events as represented by the mean threshold excess (i.e. the mean of the 5% heaviest precipitation events within 40 summers). We considered the mean threshold excess for comparison, as extreme values do not follow a normal distribution - neither block maxima nor threshold exceedances. Given the large number of excesses, the Central Limit Theorem applies, and we can thus assume a normal sampling distribution for the arithmetic mean^28^. This assumption implies that the sample means can be tested for differences employing a t-test^28^. We apply a two-sided independent samples t-test with the chosen significance levels being 90% for heavy-precipitation events and 95% for climatological summer means.

We model daily precipitation extremes based on heavy-precipitation events (i.e., threshold excesses of the 95th daily precipitation percentile) with the generalised Pareto (GP) family of distributions^29^. The GP–parameters are estimated by the maximum likelihood method. Summer extreme events are defined by the 20-season return level of daily precipitation (RL20S) in JJA. For example, the RL20S for JJA is exceeded in any JJA season with the probability 1/20, i.e., on average every 20th JJA season.

For cyclone identification and tracking we use the numerical algorithm of the P.P. Shirshov institute^30^,^31^. The algorithm is applied to 6-hourly sea level pressure (SLP) fields from the model runs. The post-processing includes filtering out cyclones with lifetimes shorter than 1 day and reaching minimum SLP over the elevated areas (>1500 m). For mapping cyclone numbers and frequencies, 6-hourly trajectories are interpolated linearly onto 10-minute time steps in order to avoid systematic and random biases^30^.

**Figure 2:**
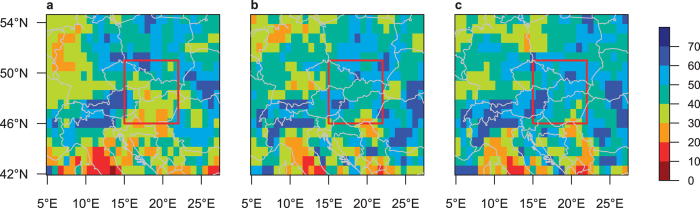
20-summer return levels in model experiments. (**a**) control, (**b**) Med_warm_, and (**c**) Glob_warm_. Red box denotes study region for area average and further analyses (15–22°E, 46–51°N). Maps created with R version 3.2.3 (www.r-project.org).

Composites of relevant variables are built on days of area-aggregated heavy-precipitation events over the study region located at 15–22°E, 46–51°N (red box in Fig. 2). To identify the synoptic pattern for flood-causing precipitation extremes in Central Europe we do not limit the analysis to single circulation types (e.g., Vb-cyclones). Instead, we apply an event-based approach associating cyclones with extreme precipitation, similar to *Pfahl and Wernli, 2012*^32^. We analyse composites of cyclone tracks passing through the study region (i.e., the cyclone centre is at least one 6-hourly time step over the study region during a heavy-precipitation event). While there is a risk that our approach might miss some cyclones causing heavy-precipitation (e.g., cyclones propagating near the study region) this methodology ensures that we exclusively consider cyclones that definitely affect precipitation in the study region.

## Results

In both the control and the Med_warm_ experiments, climatological summer mean precipitation over Central Europe (5–27°E, 42–56°N) is 2 mm/day. The Central European mean climatological summer cyclone track density is 27 cyclones per summer in both experiments (see corresponding patterns in Supplementary Figs S1–S2). The climatological summer patterns for both precipitation (compared to the E-OBS gridded observational dataset^33^) and cyclone track densities (compared to ERA-Interim reanalysis^23^,^34^) are well represented by our model (see Supplementary Information for a detailed evaluation). The Central European average of 20-summer return levels (RL20S) is 53 mm/day in the control, 55 mm/day in the Med_warm_ and 57 mm/day in the Glob_warm_ experiment (see corresponding patterns in Fig. 2). The locations of precipitation maxima that led to recent floods (e.g., eastern Austria, eastern Germany, southern Poland, Slovakia; see Supplementary Fig. S3) show high RL20S in all three experiments (Fig. 2), which gives us confidence in simulated extremes related to such synoptic situations.

**Figure 3:**
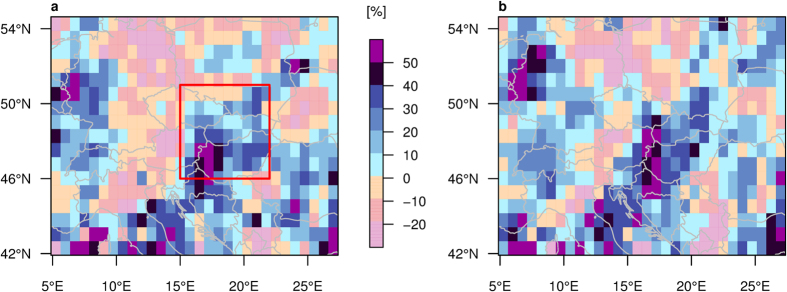
Increase in 20-summer return levels. (**a**) Med_warm_, and (**b**) Glob_warm_ experiment compared to control experiment. Red box denotes study region for area average and further analyses (15–22°E, 46–51°N). Increase of mean threshold excess (beyond 95th percentile of all summer days in the respective experiment) of box average (control: 12.3 mm/day, Med_warm_: 13.8 mm/day) is 12.2% and highly significant (p-value < 0.0005). Maps created with R version 3.2.3 (www.r-project.org).

In comparison with the control experiment, summer RL20S are amplified in Central Europe along the Vb-cyclone track in both sensitivity experiments (Med_warm_ and Glob_warm_; Fig. 3), indicating stronger precipitation extremes even though simulated changes in summer-mean precipitation are much lower (<1 mm/day, Supplementary Fig. S1). The highest increase in extreme precipitation (Med_warm_: 61.0%, Glob_warm_: 62.5%) is simulated in the Carpathian Basin; the largest decrease (Med_warm_: −37.2%, Glob_warm_: −42.1%) in northeastern Germany. Note that the highest increase is not co-located with the region of maximum precipitation on heavy events (for precipitation composites on heavy-precipitation events see Supplementary Fig. S3). The intensification of RL20S along the Vb-cyclone track can be attributed to the warmer Mediterranean Sea as it appears not only in the Glob_warm_, but also in the Med_warm_ experiment. Hence, we focus in the following on comparisons between the Med_warm_ and the control experiments. We analyse the summer season in detail as a strong intensification of precipitation extremes with a warming Mediterranean is found. In spring and autumn, however, extremes are decreasing over most of Central Europe and in regions where extremes are amplified the response is weaker than in the summer season (Supplementary Fig. S4).

To disentangle synoptic-scale events leading to severe flooding from small scale convective events with local impacts we aggregated precipitation over the study region (red box in Figs 2 and 3a). The summer RL20S averaged over this area amounts to 18.7 mm/day in the control and to 22.0 mm/day in the Med_warm_ experiment. To infer mechanisms for this increase by 17.4% we further analyse composites of relevant variables during heavy-precipitation days.

**Figure 4:**
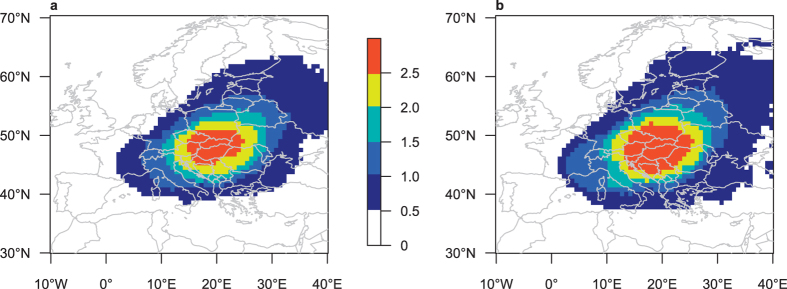
Heavy precipitation causing cyclones. Track density of cyclones that pass through the study region (15–22°E, 46–51°N; red box in Fig. 2) on days where daily precipitation aggregated over that area exceeds the 95th percentile of all summer days in the respective experiment. Counts of cyclone centres that pass within 500 km of a grid point. Unit: Cyclones per summer (JJA). (**a**) Track density in control; (**b**) Track density in Med_warm_. Maps created with R version 3.2.3 (www.r-project.org).

A considerable portion of heavy-precipitation events in both the control and the Med_warm_ experiments is associated with the passage of cyclones that originate over the Mediterranean Sea (e.g., Vb-cyclones). This is clearly indicated by track densities of cyclones that passed through the study region on event days (Fig. 4). In both experiments ~70% of the analysed heavy-precipitation events are associated with a cyclone whereof ~50% are Vb-cyclones (identified according to Supplementary Fig. S5). No significant differences in cyclone track densities and SLP composites (Supplementary Fig. S6) are found between the two experiments. Hence, neither changes in cyclone pathways or intensities nor changes in the dominant synoptic pattern can explain the simulated strengthening of extreme precipitation (note however, that we found evidence for slightly reduced cyclone dynamics in the Med_warm_ experiment compared to the control experiment, Supplementary Fig. S7).

**Figure 5:**
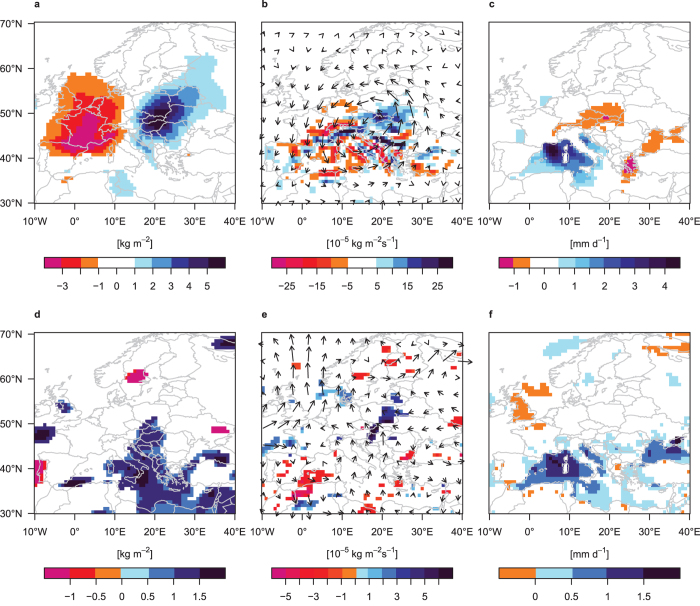
Variables related to the hydrometeorological cycle during heavy-precipitation events. Heavy-precipitation events are days where daily precipitation aggregated over the study region (15–22°E, 46–51°N; red box in Fig. 2) exceeds the 95th percentile of all summer days in the respective experiment. (**a–c**) Composite anomalies in control experiment (relative to climatology) and (**d–f**) significant differences (based on a two-sided independent samples t-test at the 90% significance level) between composites in Med_warm_ and control: (**a,d**) Column integrated precipitable water (kg m^−2^); (**b,e**) vertically integrated moisture convergence (10^−5^ kg m^−2^ s^−1^) and vertically integrated moisture transport as vectors (every fifth vector is plotted), vector length is (**b**) 50 kg m^−1^ s^−1^ per degree lon/lat and (**e**) 10 kg m^−1^ s^−1^ per degree lon/lat; (**c,f**) evaporation (mm d^−1^). Maps created with R version 3.2.3 (www.r-project.org).

Precipitable water (Fig. 5a, time lag: Supplementary Fig. S8) and moisture convergence (Fig. 5b, time lag: Supplementary Fig. S9) in the control experiment are anomalously high over Central Europe during heavy-precipitation events and on the day prior to such events. The western Mediterranean Sea is identified as a major moisture source region for heavy-precipitation in the study area by anomalous moisture divergence, moisture transport (Fig. 5b, time lag: Supplementary Fig. S9) and evaporation (Fig. 5c, time lag: Supplementary Fig. S10) on the day of and prior to the event.

Differences between heavy-precipitation-event composites of variables related to the hydrometeorological cycle in the Med_warm_ and the control experiments (Fig. 5d–f) suggest that higher Mediterranean SSTs, by further moistening the atmosphere, amplify extreme precipitation. In particular, climatological mean higher precipitable water content in the Med_warm_ experiment over the Mediterranean Sea (by 5.5% on average, Supplementary Fig. S8) is further increased on heavy-precipitation days (Fig. 5d) and the preceding day (Supplementary Fig. S8). It further extends into Central Europe where the precipitable water content is then amplified by 3–5%. This slightly exceeds the Clausius-Clapeyron rate of 7% increased saturation vapour pressure per degree of warming^35^ (see Supplementary Fig. S11). However, the higher precipitable water content is not yet sufficient to explain the intensification of heavy-precipitation events (threshold excesses) by 12.2%. Moisture convergence and transport (Fig. 5e) indicate that the extra moisture is transported from the Mediterranean Sea into Central Europe in the Med_warm_ experiment. Averaged over the study region moisture convergence is amplified by 16.2% which explains the precipitation intensification beyond the higher atmospheric moisture content (note that changes related to dynamics which may additionally contribute to amplified moisture convergence are beyond the scope of this study however). The western Mediterranean Sea is identified as the major moisture source region; this is also true for the additional moisture in Med_warm_ which is supported by the change in evaporation (Fig. 5f, time lag: Supplementary Fig. S10). The latter is amplified over the whole western Mediterranean basin synchronised with the heavy-precipitation events (western Mediterranean average: 19.9%), and also on the two preceding days, thereby strongly exceeding the climatological summer mean increase (Supplementary Fig. S10). The similarity of evaporation over land between the experiments suggests that moisture recycling is not relevant for the intensified extreme precipitation.

## Discussion

Our results show that the higher Mediterranean SSTs during recent decades amplify the magnitude of extreme precipitation events associated with cyclones that originate over the Mediterranean Sea (including those of Vb-type). The largest increase is located southeast of the precipitation maximum for both simulated heavy events and historical Vb-events. For intensified extreme precipitation changes in transients passing to Central Europe, moisture recycling or the dominant synoptic pattern are not required. The heavier precipitation events are likely related to thermodynamic changes: the stronger diabatic heating source along with increased water vapor content, intensifies moisture transport from the western Mediterranean Sea to Central Europe during heavy-precipitation events. These findings are in line with previous studies on thermodynamic changes^21^,^36^. During heavy-precipitation events the Central European region is supplied with moisture from the Mediterranean Sea which leads to precipitation increases beyond amplified saturation vapour pressure (Clausius-Clapeyron rate).

Our atmosphere-only experiments do not represent atmosphere-ocean feedbacks. We rather investigate the atmospheric response to assumed externally forced long-term SST changes. Our results are subject to the chosen atmospheric general circulation model realistically simulating the underlying processes. Yet, the major effect of increasing SSTs was thermodynamic, for which confidence is generally high compared to dynamical changes^22^. Dynamical changes were - in our model - less important for the strong precipitation response. Our study is by construction not affected by long-term internal climate variability: such macroscopic initial conditions uncertainty^37^ is caused by ocean circulation - we prescribed SSTs as climatological averages. Internal atmospheric variability is crucial to sample the distribution of extreme events. The atmosphere has essentially forgotten its initial state after a few months, such that in each year of our 40-year simulations - note that the SSTs are identical for each year - we sample from an identical climate distribution as in a 40-member ensemble of 1-year simulations. For each experiment we therefore have 184 excesses of the 95th percentile, sampled from an essentially stationary distribution. This sample is sufficient for a reasonable estimate of 20-season return levels^38^. We found a highly significant amplification of area-averaged heavy-precipitation events (excesses of the 95th percentile of all days in the respective experiment) with a warmer Mediterranean by 12.2%. With unchanged variance in both samples an increase by only 5.3% would be sufficient for a significant response at the 95% significance level, which gives us confidence in the simulated intensification of precipitation.

Amplified Mediterranean SSTs are both forced anthropogenically^20^ and related to internal decadal-scale variability (e.g., Atlantic Multidecadal Oscillation)^39^. Increased subsidence in the descending branch of the Hadley circulation, associated with anticyclonic conditions over Central Europe may also contribute to warm anomalies in the Mediterranean Sea, and suppress cyclonic activity^40^. Yet, climate change scenarios show only slight reductions in Central European summer cyclone track densities^24^ and Vb-cyclones^14^, suggesting that such cyclones will most likely still be relevant in a future climate. Our atmosphere model sensitivity experiment suggests that the projected intensification of precipitation related to Vb-cyclones^14^,^26^ can be attributed to the rise in Mediterranean SSTs which is itself projected to continue throughout the 21st century^20^.

## Additional Information

**How to cite this article**: Volosciuk, C. *et al*. Rising Mediterranean Sea Surface Temperatures Amplify Extreme Summer Precipitation in Central Europe. *Sci. Rep*. **6**, 32450; doi: 10.1038/srep32450 (2016).

## Supplementary Material

Supplementary Information
