# Supplementary material for: Rising Mediterranean Sea Surface Temperatures Amplify Extreme Summer Precipitation in Central Europe
Source: Sci Rep. 2016 Aug 30;6:32450. doi: 10.1038/srep32450 (PMC5004138; doi:10.1038/srep32450)
Supplement: Supplementary Information [file srep32450-s1.pdf]

Rising Mediterranean Sea Surface Temperatures Amplify Extreme Summer Precipitation in Central Europe
SUPPLEMENTARY INFORMATION

Claudia Volosciuk*[1], Douglas Maraun[1,2], Vladimir A. Semenov[1,3,4], Natalia Tilinina[5], Sergey K. Gulev[5] & Mojib Latif[1]

Content

- Evaluation of Model Experiments
- Table S1
- Figures S1–S11; in all Figures:
  - Heavy precipitation events are days on which daily precipitation aggregated over 15 – 22°E, 46 – 51°N (red box in Fig. 2 in the main paper) exceeds the 95th percentile of all summer days in the respective model experiment.
  - Significance is tested based on a two-sided independent samples t-test. Differences between composites on heavy precipitation events (summer mean climatologies) are tested at the 90% (95%) significance level.

Evaluation of Model Experiments

Summer mean precipitation in our ECHAM5 model experiments (Fig. S1a, b) is evaluated with the European daily high-resolution (0.25° × 0.25°) gridded dataset (E-OBS, version 10) of precipitation[1] (Fig. S1c), developed in the framework of the ENSEMBLES project. E-OBS at 0.25° was averaged to the resolution of the model simulations by area conservative remapping. As summer mean precipitation climatologies in our sensitivity experiments (Fig. S1a, b) are very similar (Fig. S1d) we compare both experiments to the E-OBS precipitation climatology over both forcing periods, i.e., 1970 – 2012 (Fig. S1c). The precipitation pattern over Europe is well captured in both experiments. Major deficiencies are a wet bias at the Scandinavian west coast and a dry bias in eastern Austria. Central and Eastern Europe are slightly wetter in the experiments than in the observations. These differences are small, and the overall pattern is well captured. The bias is very low for both experiments (Tab. S1).

We did not evaluate simulated 20-summer return levels with the E-OBS dataset as the rain gauge density in E-OBS is sparse in some regions, especially in Eastern Europe[1]. Hence, extreme precipitation is likely to be underrepresented in these regions by the E-OBS dataset, and differences between the model simulations and observations are therefore not necessarily attributable to model deficiencies.

Summer mean cyclone track densities in our experiments (Fig. S2a, b) are evaluated with ERA-Interim reanalysis[2,3] (Fig. S2c). The summer cyclone track pattern is well captured over Europe in both experiments. ECHAM5, however, generally simulates more cyclones than ERA-

Interim. This has already been reported for ECHAM5 simulations compared with ERA40[4]. Although ECHAM5 generally simulates more cyclones *Nissen et al., 2013*[4] found the share of Mediterranean cyclones developing into Vb-cyclones to be similar in coupled ECHAM5 scenario simulations and ERA40. In our experiments, the Mediterranean storm track is also stronger than in reanalysis (ERA-Interim in our case) with the strongest bias located over Turkey. The bias of the European mean is $\sim$4 cyclones per summer in both experiments (Tab. S1).

Table S1: **Bias of summer mean precipitation and cyclone tracks in model experiments.** Error of simulated European mean (10°W–40°E, 30–70°N) and root mean squared error (RMSE) of the pattern for JJA mean precipitation (mm/day) and JJA mean cyclone track density (cyclones per summer) in the ECHAM5 control and $\mathrm{Med}_{\mathrm{warm}}$ experiments evaluated against the E-OBS precipitation gridded dataset and cyclone tracks from ERA-Interim reanalysis

	European mean error		Pattern-RMSE	
	Control	$\mathrm{Med}_{\mathrm{warm}}$	Control	$\mathrm{Med}_{\mathrm{warm}}$
Precipitation	0.26	0.29	0.009	0.007
Cyclone track density	4.02	4.22	0.08	0.08

1. Haylock, M. R. *et al.* A European daily high-resolution gridded data set of surface temperature and precipitation for 1950–2006. *Journal of Geophysical Research* **113**, D20119 (2008).

2. Dee, D. P. *et al.* The ERA-Interim reanalysis: configuration and performance of the data assimilation system. *Quarterly Journal of the Royal Meteorological Society* **137**, 553–597 (2011).

3. Tilinina, N., Gulev, S. K., Rudeva, I. & Koltermann, P. Comparing cyclone life cycle characteristics and their interannual variability in different reanalyses. *Journal of Climate* **26**, 6419–6438 (2013).

4. Nissen, K. M., Ulbrich, U. & Leckebusch, G. C. Vb cyclones and associated rainfall extremes over Central Europe under present day and climate change conditions. *Meteorologische Zeitschrift* **22**, 649–660 (2013).

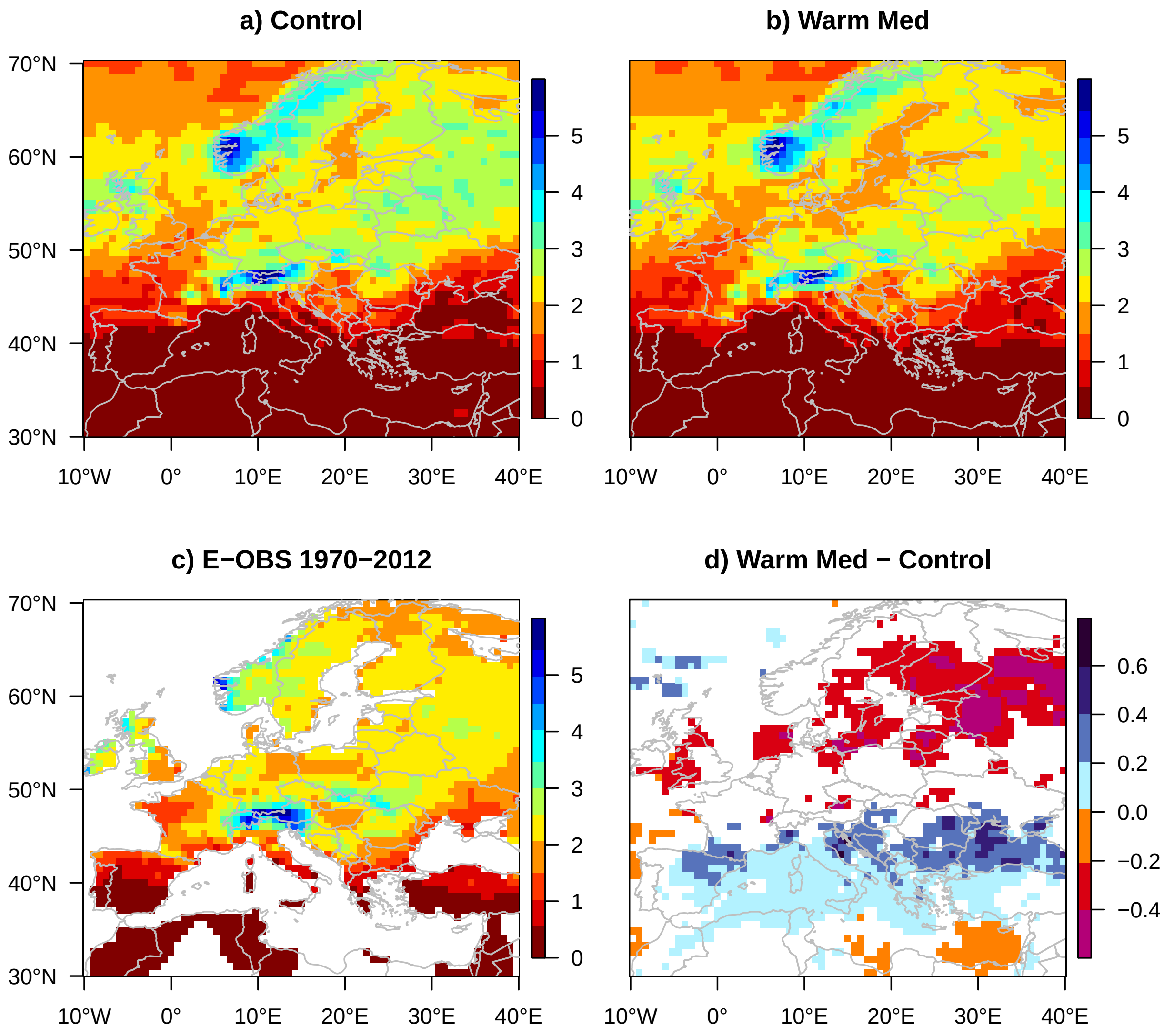

Figure S1: **Summer mean precipitation.** Mean JJA precipitation (mm/day) in (a) control experiment, (b) warm Mediterranean (Med_{warm}) experiment, and (c) E-OBS, version 10. (d) Significant differences between JJA-climatology in Med_{warm} and control. Maps created with R version 3.2.3 (www.r-project.org).

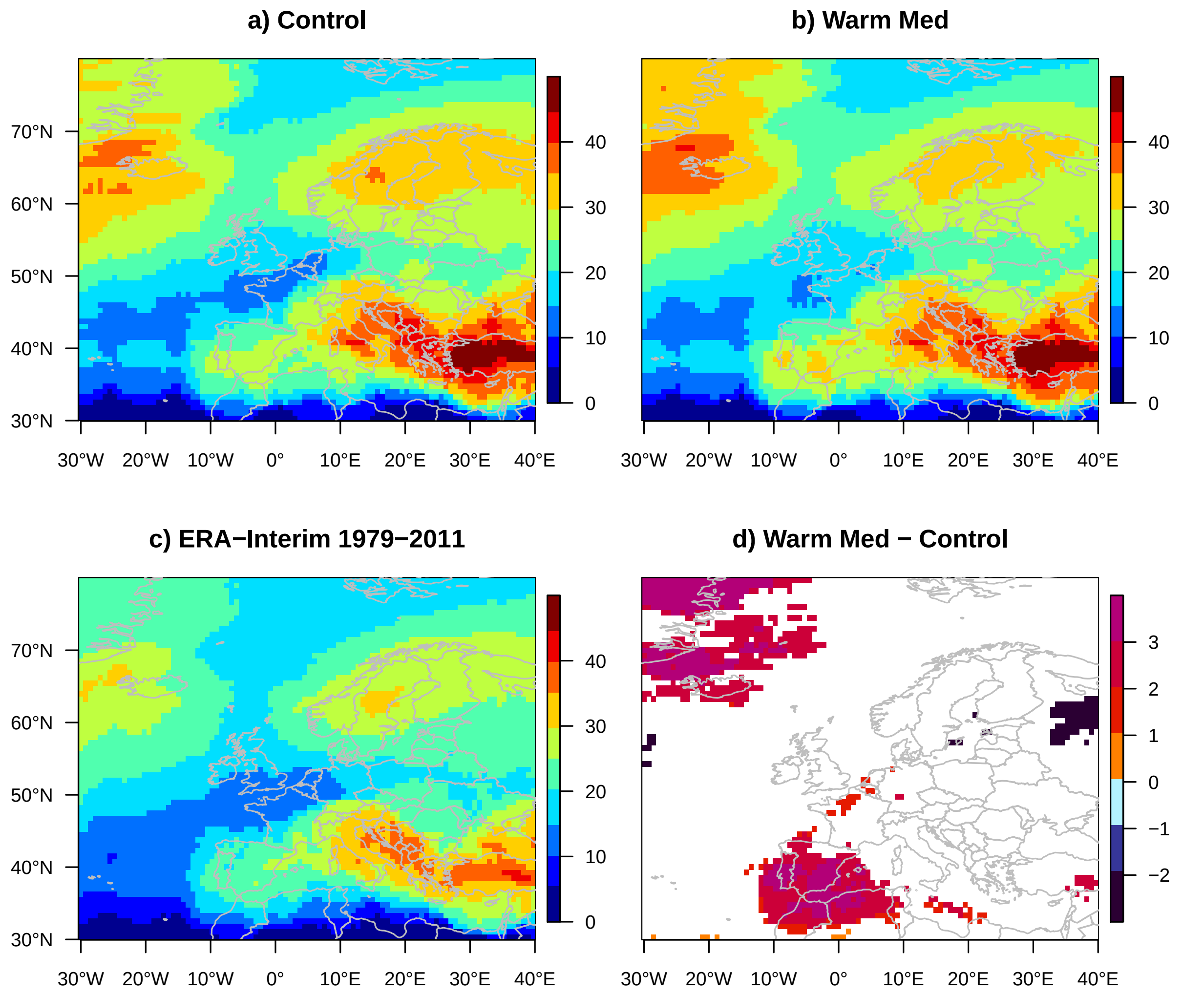

Figure S2: **Summer cyclone track densities.** Mean JJA cyclone track densities (cyclones per summer) in (a) control experiment, (b) Med_{warm} experiment, and (c) ERA-Interim. Counts of cyclone centres that pass within 500 km of a grid point. (d) Significant differences between JJA-climatology in Med_{warm} and control. Maps created with R version 3.2.3 (www.r-project.org).

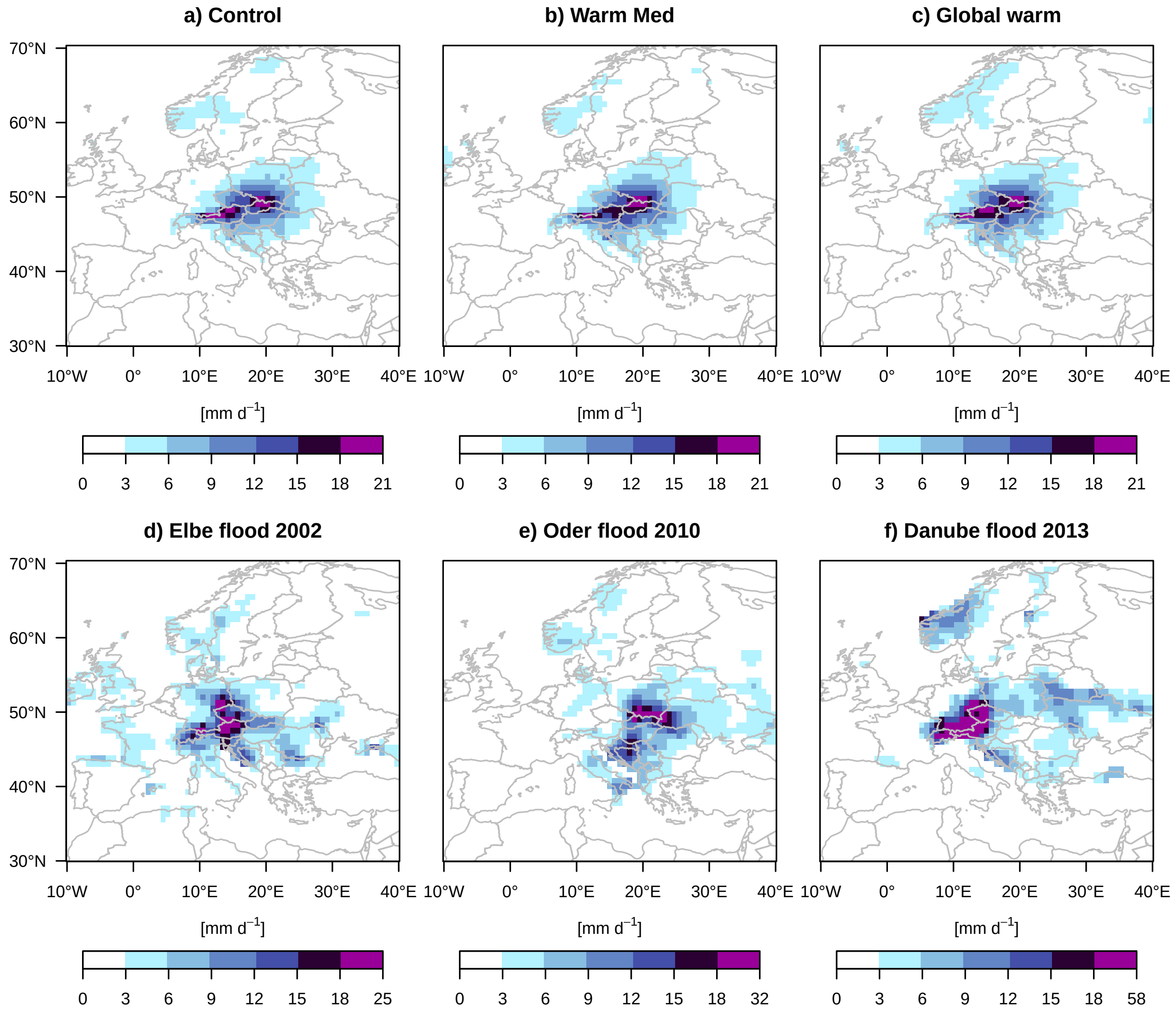

Figure S3: **Precipitation on heavy-precipitation events.** (a – c) Composites of daily precipitation on heavy-precipitation events in model experiments. In all experiments precipitation maxima on heavy-precipitation events are in Austria and Slovakia. (a) Control, (b) $\mathrm{Med}_{\mathrm{warm}}$, and (c) $\mathrm{Glob}_{\mathrm{warm}}$. (d – f) Observed (E-OBS, version 10) aggregated precipitation on recent heavy-precipitation events caused by Vb-cyclones that led to severe flooding, normalised by the number of event days. (d) Elbe 2002, (e) Oder 2010, and (f) Danube 2013. Note the different colour scales. Composites (a – c) represent an average of several events where single peaks are smoothed, and can thus not directly be compared to single cases (d – f). Nevertheless, the affected regions by the three cases are similar to the composites of our model experiments. Note that the study region for the heavy events in the composites constrains the affected region. Maps created with R version 3.2.3 (www.r-project.org).

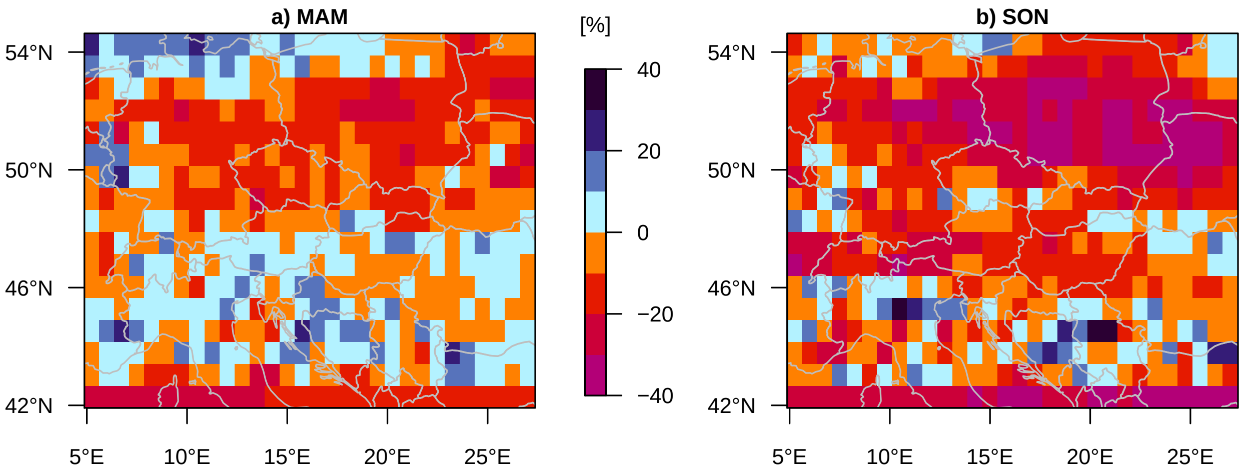

Figure S4: **Changes in 20-season return levels.** $\mathrm{Med}_{\mathrm{warm}}$ compared to control. (a) Spring (March-April, MAM), and (b) autumn (September-November, SON). Maps created with R version 3.2.3 (www.r-project.org).

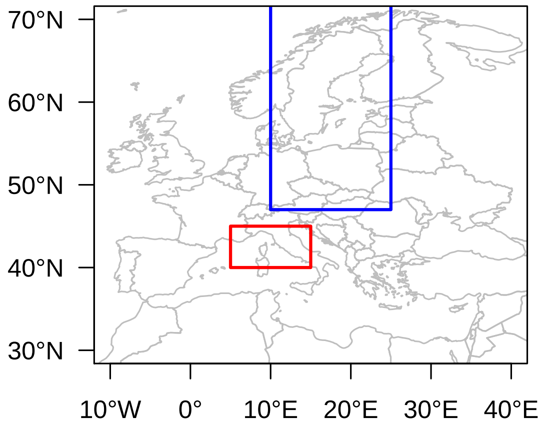

Figure S5: **Identification of Vb-cyclones.** Cyclone tracks are identified as Vb-cyclones if they fulfill the following criteria: (1) cyclone must pass the typical Vb-cyclogenesis area (5 – 15°E & 40 – 45°N, red box), (2) cyclone must cross 47°N (horizontal blue line) between 10–25°E (vertical blue lines). Map created with R version 3.2.3 (www.r-project.org).

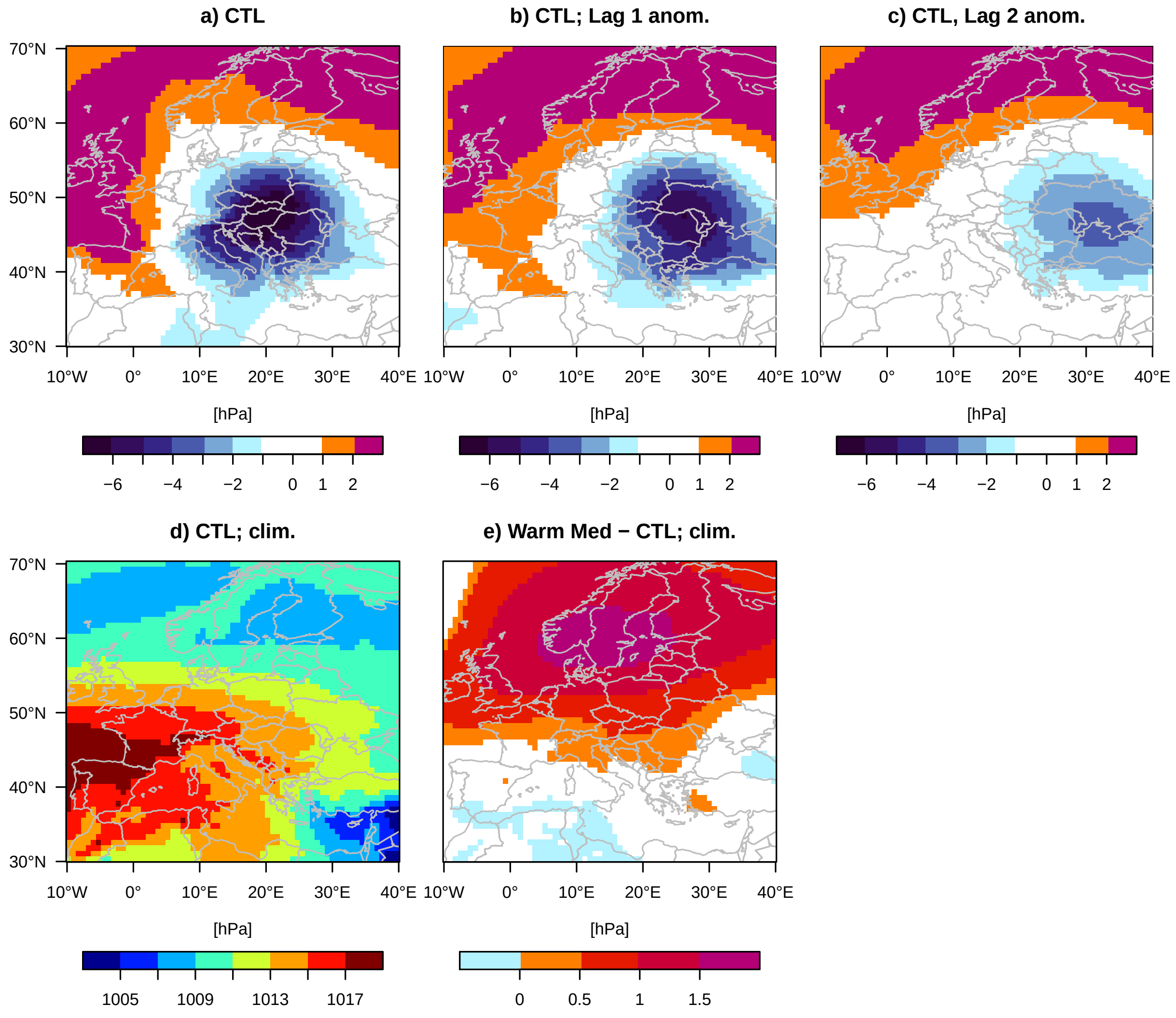

Figure S6: **Sea level pressure.** (a – c) Composites of mean sea level pressure (hPa) anomalies (relative to climatology) in the control experiment on heavy-precipitation events and on the two preceding days. No significant differences between composites from the $\mathrm{Med}_{\mathrm{warm}}$ and control experiments were found. (d) JJA-climatology in the control experiment. (e) Significant differences between JJA-climatology in $\mathrm{Med}_{\mathrm{warm}}$ and control. Maps created with R version 3.2.3 (www.r-project.org).

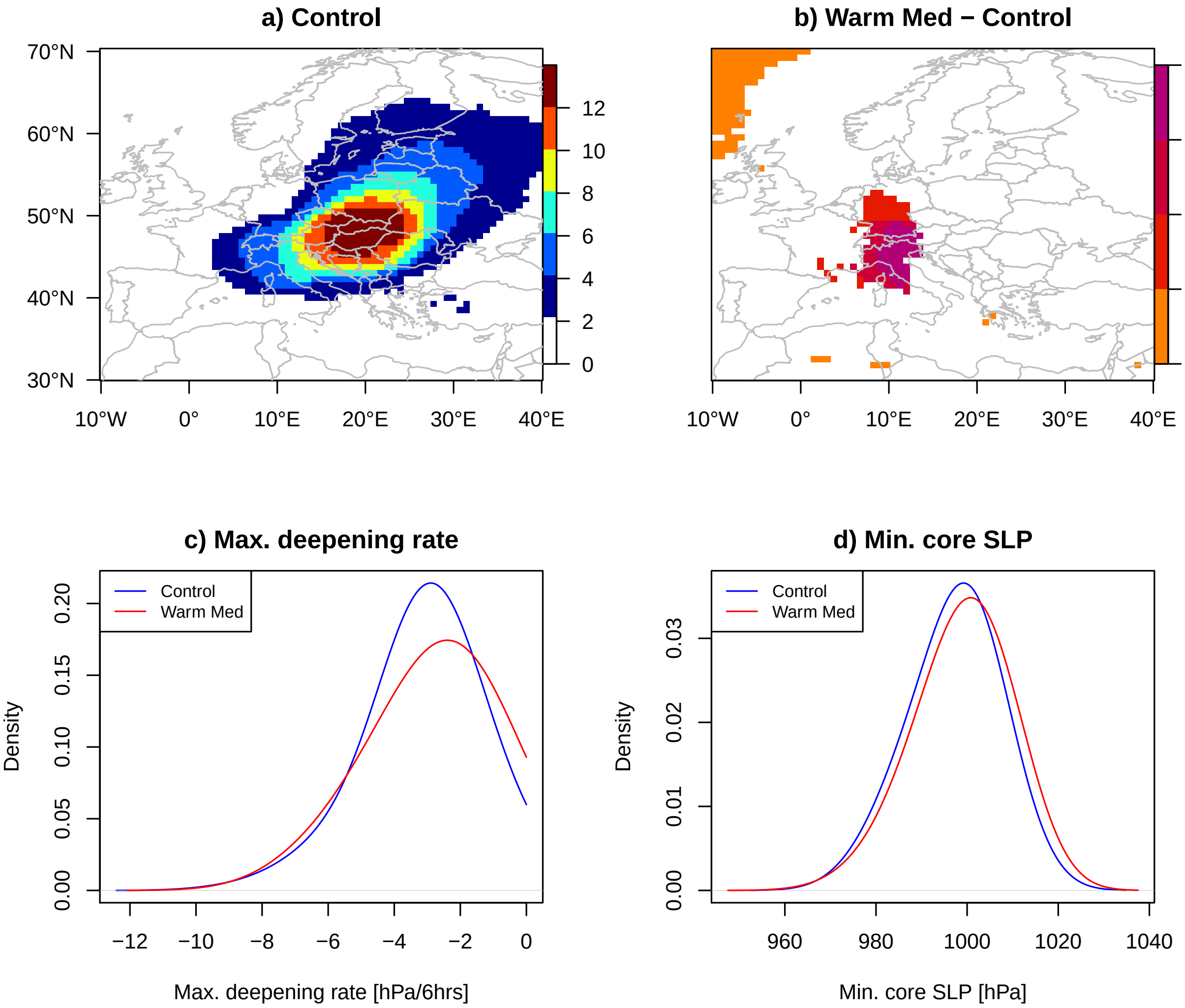

Figure S7: **Reduced dynamics of heavy-precipitation causing cyclones.** Cyclone tracks passing over 15 – 22°E, 46 – 51°N (red box in Fig. 2 in the main paper) on heavy-precipitation events are considered. (a) Summer (JJA) cyclone frequency in control experiment. Counts of cyclone centres that pass within 500 km of a grid point. Unit: 6-hourly time steps per summer. (b) Significant differences between cyclone frequency in Med_{warm} and control, indicating slower travelling cyclones in Med_{warm} experiment. (c) Density of cyclone-centre maximum deepening rate (hPa/6hrs), indicating slower deepening of cyclones in Med_{warm} experiment. (d) Density of minimum core pressure (hPa), indicating slightly less deep cyclones in Med_{warm} experiment. Maps created with R version 3.2.3 (www.r-project.org).

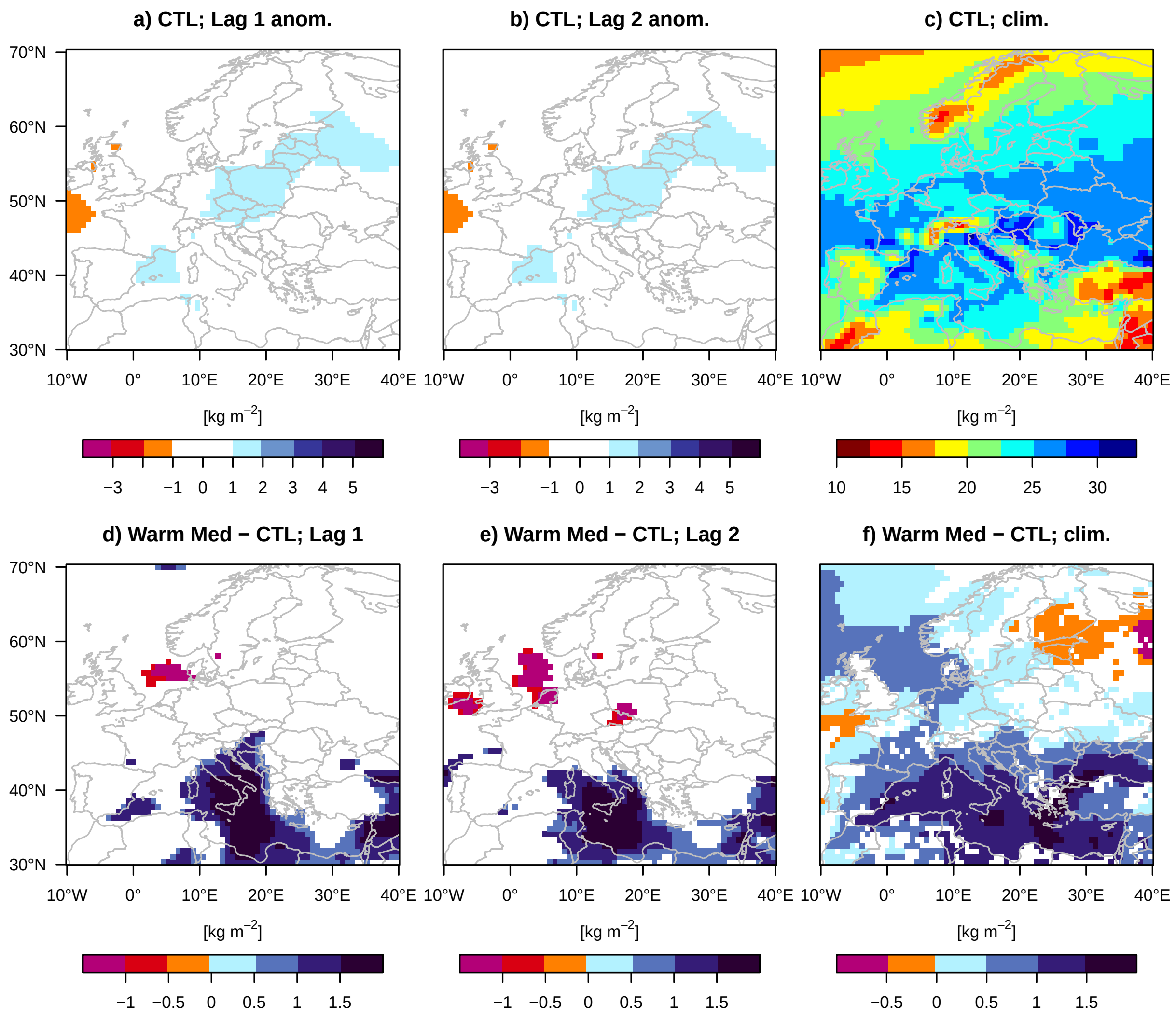

Figure S8: **Precipitable water.** Composites of column integrated precipitable water (kg m^{-2}) on (a, d) lag 1 and (b, e) lag 2 days before heavy-precipitation events: (a, b) anomalies in control experiment (relative to climatology) and (d, e) significant differences between composites in Med_{warm} and control. (c) JJA-climatology of control experiment. (f) Significant differences between JJA-climatology in Med_{warm} and control. Maps created with R version 3.2.3 (www.r-project.org).

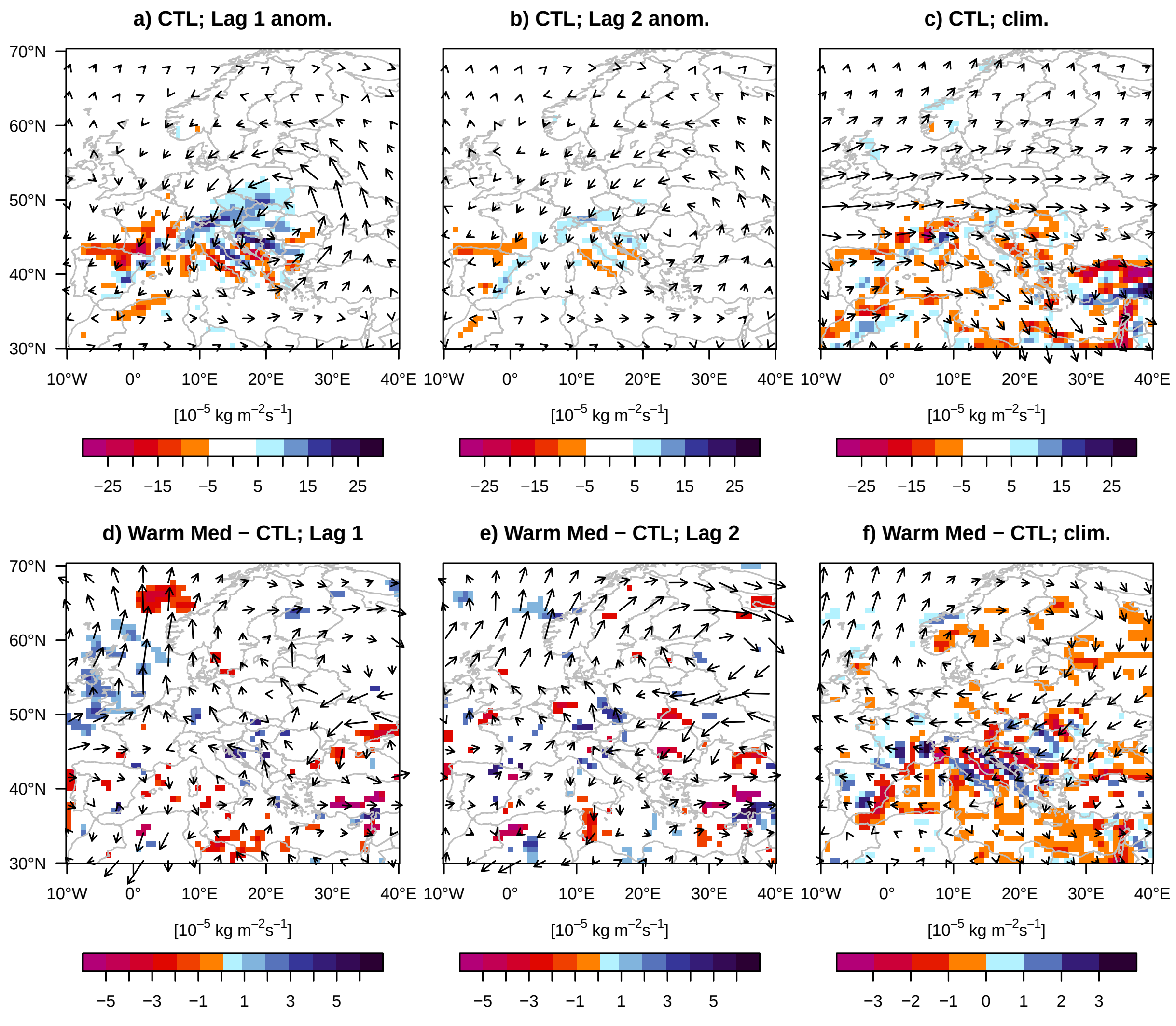

Figure S9: **Moisture convergence and moisture transport.** Same as Fig. S8 but for vertically integrated moisture convergence (10^{-5} kg $m^{-2}s^{-1}$) and vertically integrated moisture transport as vectors (every fifth vector is plotted), vector length is (a - c) 50 kg $m^{-1}s^{-1}$ per degree lon/lat and (d - f) 10 kg $m^{-1}s^{-1}$ per degree lon/lat. Maps created with R version 3.2.3 (www.r-project.org).

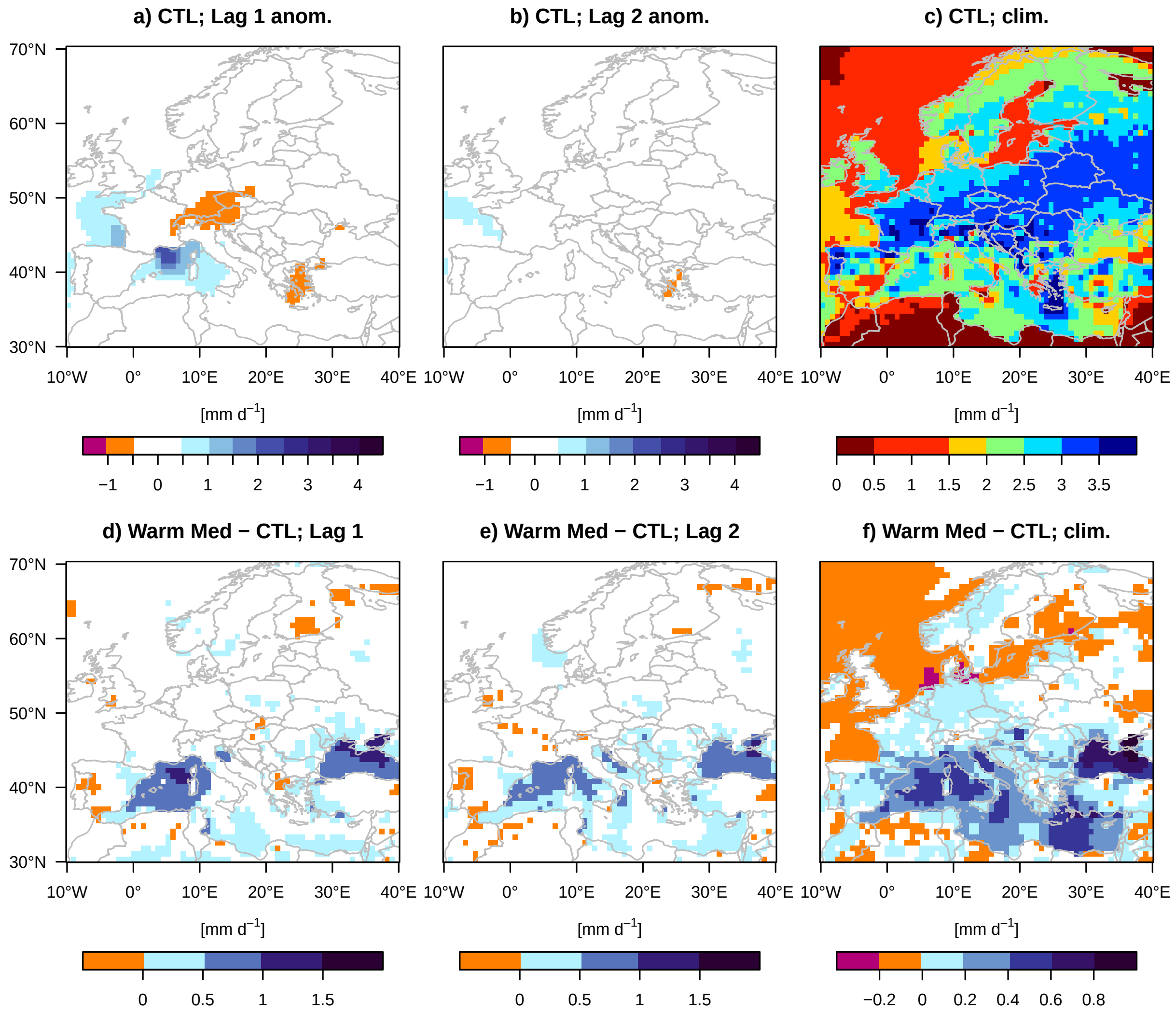

Figure S10: **Evaporation.** Same as Fig. S8 but for evaporation (mm d^{-1}). Maps created with R version 3.2.3 (www.r-project.org).

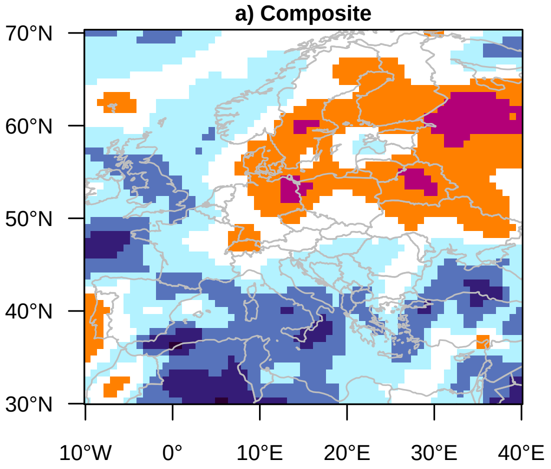

Figure S11: **Specific humidity increase beyond Clausius-Clapeyron rate.** Difference (%) between vertically integrated (up to 200 hPa) specific humidity in $\mathrm{Med}_{\mathrm{warm}}$ experiment and the Clausius-Clapeyron related amplification (7% per degree of warming) of the same quantity in the control, based on the temperature difference between $\mathrm{Med}_{\mathrm{warm}}$ and control. 0% would be the specific humidity increase based on Clausius-Clapeyron. (a) Composite of heavy-precipitation events, and (b) climatological summer (JJA) mean average. Maps created with R version 3.2.3 (www.r-project.org).
